# Contracting with sequential care providers

**DOI:** 10.1186/s13561-024-00572-w

**Published:** 2024-12-19

**Authors:** Sverre Grepperud, Pål Andreas Pedersen

**Affiliations:** 1 University of Oslo, Oslo, Norway; 2 Nord University Business School, Bodø, Norway

**Keywords:** Vertical relations, Coordination, Sequential production, Semi-altruistic agents, H44, H51, I18, J38 and L33

## Abstract

**Background:**

The literature on care coordination refers to high service costs, low quality, and consumer dissatisfaction, as the consequences of institutional fragmentation and uncoordinated care.

**Objectives:**

In this work we are concerned with the role financial incentives (reimbursement schemes) might play in promoting coordinated care when providers are organized sequentially along a care pathway and the clients (patients) are transferred from one caregiver to another.

**Methods:**

We apply a game-theoretic framework to analyze the situation where three providers provide services to a patient group and there are interdependencies between the providers in terms of cost-externalities and altruistic patient preferences.

**Results:**

For activity-based contracts, the incentives for cost containment are efficient (internal efficiency), while the incentives for quality provision are inefficient due to preference misalignments and poor coordination that derive from funding costs, imperfect altruism, the presence of externalities and strategic behavior. The optimal cost-based contracts are mixed contracts that vary across providers according to their position in the production chain, and they consist of the following three elements; (i) fixed budgets, (ii) payments contingent upon the treatment costs of production chain followers (integrated penalties), and (iii) payments contingent upon the providers’ own treatment costs (positive or negative cost-sharing). For these contracts, the providers are typically internally inefficient, while the inefficiencies associated with preference misalignments and poor coordination are solved.

**Conclusions:**

Our production chain perspective, when compared to single-provider approaches, enhances the appeal of cost-based contracts relative to pure prospective contracts.

## Introduction

Groups of consumers of social care and health care services typically need services from multiple providers. For social care services, clients may need economic support, housing services, vocational training and child welfare support.^1^ For health care services, patients may need assistance from primary care physicians, specialized healthcare institutions, rehabilitation centers and nursing institutions (home care or institutional care).^2^ This means that care providers are typically organized sequentially (value generating chains) where clients, depending on their needs, are transferred from one caregiver to another [30, 73]. In this perspective, the contribution from one provider becomes the extension of provisions of others, suggesting the presence of interdependencies. On the other side, the standard organization of care pathways consists of loosely connected care providers since being independent, with their own management structures, and since being reimbursed separately. This type of institutional fragmentation is believed to induce coordination problems [80]. 

Coordination represents ways of managing interdependencies both within and between organizations and can be achieved through a variety of means. At the micro level, mechanisms such as routines, standardization, meetings, and information systems can be applied to improve coordination [29]. At the meso-level, centralization (consolidation) and delegation are strategies that may prevent fragmented care. Centralization typically involves ownership reforms in that organizations, formerly being separate, now become integrated into one entity. Delegation, on the other hand, implies some type of authority transfer from the centralized level to the decentralized level. A third avenue for improved coordination is the use of payment incentives (reimbursement). In this work we are concerned with the role reimbursement systems might have in coordinating services among multiple providers that act in sequential production chains. Specifically, we focus on how pure prospective pay performs relative to contracts that utilize information on ex-post treatment costs (retrospective pay). Since the mid-1908s, the health economic literature has been concerned with discussing the advantages and disadvantages of retrospective vs. prospective schemes.^3^

The literature on care coordination refers to high service costs, low quality, and consumer dissatisfaction, as consequences of institutional fragmentation. To improve the delivery of care services, an increasing number of initiatives are implemented to meet the demand for coordinated services, both within (primary, secondary, and tertiary care) and across sectors (health care, social care and education). These initiatives represent a wide range of approaches, and they appear under headings such as integrated care, value-based care, integrated funding and new innovative payments.^4^ One example is when a group of providers align their resources (possibly, in combination with joint monitoring). There are also examples of more structural integration initiatives in the sense that the funding streams do not remain entirely separate [66].^5^ This is the case when sponsors earmark funds meant to support coordination or when sponsors contracts with a group of providers rather than with each of them separately (the pooling of budgets).

An example of a coordination problem that has attracted considerable attention is that patients in need of care services stay in hospitals even when ready for discharge because they are waiting for admission to long term care (bed blocking). This problem is believed to arise from the organizational structure where nursing homes and home care are the responsibility of the municipalities while hospital care is the responsibility of trusts. The introduction of financial penalties in such situations has been shown to lead to a large reduction in the frequency of bed-blocking [56]. In England, the Department of Health organizes health care at a national level, while the social care system is organized at the regional level, under the control of local authorities. This organization is believed to make patient navigation between the systems complex as well inducing unnecessary hospitalizations for people with long term conditions and social care needs [34]. In the Netherlands, the GPs act as gatekeepers to secondary care, but this system has been blamed for providing an insufficient service quality especially for those with chronic conditions as well as causing a high growth in healthcare expenditures because of too many referrals [8, 51]. In Sweden the hospitals have a catchment area responsibility meaning that the patient choice is limited. Furthermore, since hospital reimbursement relies on Diagnosis Related Groups (DRGs), providers have a limited financial responsibility. For surgical procedures, this means that the hospitals are not responsible for the posthospital period. In the region of Stockholm, a reform was introduced by conditioning the payments for hip replacements surgeries on resource use, quality (adverse events) and patient satisfaction. This expanded responsibility led to positive outcomes, including shorter lengths of stay and lower rates of adverse events, indicating that financial incentives which hold providers accountable for the entire care cycle may be effective [35].

A number of reviews, concerned with the effects of integrated care and integrated funding, consider both intersectoral interventions [5, 45, 66, 68, 69, 78, 82] and intra-sectoral interventions [5, 13, 47, 65, 71].^6^ However, despite of some primary studies reporting about positive results in terms of improved perceived care quality, increased client satisfaction and improved access to care, the evidence is mixed and characterized by methodological limitations and poor intervention descriptions, making it difficult to draw valid and reliable conclusions. Furthermore, there is a shortage of analytical (theoretical) studies in the literature evaluating the potential role of contracts in promoting coordination. Such insights would be valuable for policymakers in designing effective payment schemes. In this work, we aim to contribute to this knowledge.

We present a model that studies quality and cost containment incentives where the providers have client-regarding preferences (partial altruism), hence quality becomes agency-driven (a supply-side perspective). Furthermore, interdependencies appear to be inevitable (natural) characteristics of environments with a sequential structure, and here we study outcome interdependencies that arise because of provider altruism in combination with client benefits being a function of the qualities provided by the chain partners (joint outcome). Cost interdependencies follow because the quality provided by a given provider impacts the production costs of the production chain followers. It is assumed that the joint outcome (client benefits), provider qualities and cost containments efforts are non-contractable, while the number of clients and service costs are contractible.

The principal, here termed the sponsor, acts as a social planner having a benefit function representing consumers’ preferences. When deciding on the payment rule, the sponsor considers the effects on the incentives for cost containment, quality, and client group size (admittance). Our model is meant to mirror a public system with publicly owned providers that have separate management structures and catchment area responsibilities being funded by general taxation (centralized funding).^7^ Furthermore, our analysis relates to the literature on moral hazard in multi-level organizations and the literature concerned with the contracting of health care providers.

Works on multi-level organizations are concerned with issues such as; (i) collusion [3, 18, 63], (ii) whether the actions of leaders should be observable to followers or not [15, 70, 90], (iii) integration versus separation – whether a single agent (integration) or two different agents (separation) should be in charge of the productive stages [76, 81] and (iv) optimal contracts when the joint output is verifiable [79, 89]. Strausz [79] and Winter [89] consider non-altruistic agents that move sequentially and where the agents observe the decisions of their predecessors. Strausz [79] allows for cost-interdependencies and finds that a balanced budget-sharing rule achieves social efficiency. The equilibrium strategies of the agents are not to shirk in the absence of any shirking of predecessors and shirk if observing shirking predecessors. Winter [89] assumes cost-independence and agents that make binary investment decisions (invest or not invest), where all must invest for the project to become successful. The main conclusion is that the late movers must be provided with additional incentives (higher rewards), relative to the early movers, because they are observed by a smaller number of agents (a lower implicit threat against shirking).

Relevant works on the contracting of health care providers, for non-contractible quality, are Ellis and McGuire [24, 25], Chalkley and Malcomson [16], and Jack [48]. Ellis and McGuire [24, 25] find, when a provider values patient benefits as the social planner (perfect agents), that the welfare-optimal quality level will be provided. If the valuation is less (imperfect agents), however, an under-provision of quality follows, and this inefficiency is corrected by introducing cost-sharing contracts (welfare-optimal contracts). Chalkley and Malcomson [16] consider costs that depend on quality and cost-containment effort,thus cost-sharing becomes costly since reducing cost-containment incentives (second-best contracts). Jack [48] considers providers that vary with respect to the degree of agency and derive optimal contracts when neither provider characteristics nor quality are observable. Other studies on quality incentives, where quality is driven by demand rather than agency, include Ma [62], Rogerson [75], and Chalkley and Malcomson [17]. There is also a literature on quality competition in oligopolistic health care markets with regulated prices. Typically, this literature identifies a positive relationship between competition and quality.^8^ An exception is Brekke et al. [9], where this relationship is generally ambiguous.^9^

Two of our main assumptions will be commented upon in more detail. First**,** the providers are assumed to have client-regarding preferences (semi-altruism) meaning that they, to some extent, care about client benefits (altruistic concerns). This assumption draws upon the literature on non-pecuniary motivations such as intrinsic motivation, public service motivation and pro-social behavior (see e.g., [6, 20, 31]). Francois and Vlassopoulus [28] distinguish between action-oriented motivations (impure altruism), where agents obtain a direct benefit from the effort expended into a task (“warm glow”) and outcome-oriented motivations, where agents care about the overall value of the good to which they contribute (pure altruism).

The assumption of altruistic care providers is common in the theoretical literature of health economics. In addition to the works of Ellis and McGuire [16, 24] and Jack [48], similar assumptions are present in Eggleston [22], Heyes [43], Chone and Ma [19], Kaarboe and Siciliani [50] and Kristensen et al., [54].^10^ There is also an empirical literature on physician altruism (patient-regarding preferences) that applies behavioral data from controlled settings (experimental designs). Hennig-Scmidt et al., [41] find that patients’ health benefits, in addition to payment incentives, are of considerable importance. Similar conclusions are available from Henning-Scmidt and Wiesen [42], Kesternich et al., [53] and Brosig-Koch et al., [10, 11]. Godager and Wiesen [33] and Wang et al., [86] quantify the degree of altruism by estimating the weight attached to health benefits. Wang et al., [86] use a dataset consisting of three subject pools (Chinese medical students, German medical students and Chinese medical doctors) to estimate a measure of the relative weight of patient benefits.^11^ Godager and Wiesen [33] apply a dataset comprised of 42 medical students and find that almost all medical students put a positive weight on patients’ health benefits. The majority, either attaches equal weights to profit and health benefits (29%) or puts an even higher weight on health benefits (44%), but there is considerable variation across laboratory participants.^12^

Second, it is assumed that the qualities supplied by the chain members (partners) have implications for downstream costs (cost externalities). This means that a higher quality from a given provider implies that less resources are needed at the next stage of the production chain. Such interdependencies seem to be confirmed by the literature. Fortney et al. [27] identifies a substitution effect between primary care and specialist encounters in the USA. Forder [26], studying utilization of social care (home care services) and the demand for hospital services in England, finds that hospital services and care services for older people are substitutes (at the margin). Multidisciplinary rehabilitation is found to reduce the length of stay in acute care and reduce the need for continued care [21] and [83]). A systematic review by van Hoof et al., [84] finds, for 11 out of 14 studies, that primary care interventions substitute for outpatient hospital care. Lau et al., [57] find that community care in England acts as weak substitutes with all hospital activities and that primary care contacts are weak substitutes for emergency attendances and admissions.^13^

The main findings of this analysis are summarized below. First, pure prospective pay (price per client and fixed budgets) ensures internal efficiency for all production chain members, however, such contracts do not address preference misalignments, and they give rise to coordination problems which in sum produce suboptimal quality levels and a suboptimal allocation of quality across the production chain. Second, under prospective pay, the number of included clients can be too high or too low, relatively to the welfare optimal level. Third, when allowing for contracts that are based upon provider treatment costs (retrospective elements), it is shown that mixed contracts, containing fixed budgets, cost-sharing and integrated penalties (penalties being contingent upon the costs of subsequent providers),^14^ eliminate the problems identified under pure prospective pay. Fourth, the mixed contracts vary across chain partners depending on their position in the production chain, but these contracts will not attain the welfare-optimal solution when the mixed rule advocates the use of cost-sharing. Fifth, if the welfare loss that follows from practicing fixed prices and fixed budgets, stemming from preference misalignments and non-coordinated care, is higher (lower) than the welfare loss that follow from practicing cost-based schemes, stemming from production inefficiencies, the cost-based (pure prospective) contracts are preferred from a welfare point of view. Sixth, the first-best solution can only be attained if all providers are perfect agents, since now the use of cost sharing to steer quality becomes unnecessary. Over the last three decades there has been a gradual shift in the reimbursement rules for health care providers in favor of prospective funding (payments per patient or Diagnosis Related Groups). Our analysis suggests that if uncoordinated care is a significant problem, the introduction of retrospective elements into the contracts (cost-based contracts) may be welfare-improving since having a potential to reduce coordination problems.

This paper is organized in the following way. “The model and the first best solution” section presents the model and derives the welfare optimal conditions. In “Prospective contracts” section, we study a sponsor that contracts with each provider by means of pure prospective contracts (price per client and/or fixed budgets). “Cost-based contracts” section analyzes cost-based contracts (retrospective contracts), “Discussion” section discusses our findings, while “Conclusion” section provides some concluding remarks.

## The model and the first best solution

In the following, we study a sponsor and three providers that act as partners in the production of services to a group of clients. The model is abstract in the sense that we do not include client characteristics such as problems in everyday living, unemployment, health state or severity. We abstract from such dimensions to focus on the relevance of interdependencies that take place between sequentially organized providers. As an illustrative example, one may think of a patient seeking services from a primary care center (upstream provider). After the examination, the patient is transferred to specialist care for further services (midstream provider). When the specialized treatment is completed, the patient is transferred to a rehabilitation institution (downstream provider). In this way, all three providers contribute to producing client welfare.

It follows from our set-up that the number of clients included is selected by the upstream provider, and then, after receiving upstream services, these clients are transferred to the downstream partners. The model consists of four stages (time periods) and the time sequence is assumed to be as follows. In the first period, the sponsor announces the payment contracts. In the second period, the upstream provider decides on quality (*X*), the number of clients *(n*), and cost-containment effort $$\left(e\right).$$ In the third period, the midstream provider decides on quality (*Y*) and cost-containment effort ($$f)$$, and finally, in the fourth period, the downstream provider decides on quality (Z) and cost-containment effort ($$g$$).

The benefit function, *V(X, Y, Z, n)*, is a function of the quality supplied by each chain member and the number of clients, and we introduce the following assumptions ${V}_{X}>0, {V}_{Y}>0, {{V}_{Z}>0, {V}_{n}>0,V}_{XX}<0, {V}_{YY}<0, {V}_{ZZ}<0$ and ${V}_{nn}<$ 0. Firstly, this means that the sponsor (or client) values a higher quality level from each provider, but the marginal valuation decreases with a higher quality level. Secondly, to simplify, the ranking of the clients for all quality mixes, (*X,Y,Z*) is defined in such a way that a higher number of clients is valued positively by the sponsor, but this increase becomes lower for an increasing number of clients.^15^

The altruistic preferences follow from the benefit function, where $\alpha V$ (*X,Y,Z,n)* is upstream preferences and $\alpha$ the degree of upstream altruism. The midstream and downstream preferences are $\beta V$ (*X,Y,Z,n)* and $\gamma V$ (*X,Y,Z,n)*, respectively, where $\beta$ ($\gamma$) is the degree of altruism held by the midstream (downstream) provider. In the following, we restrict out attention to providers that only partially include client benefits, thus we have that; $0<\alpha <1$, $0<\beta <1$ and $0<\gamma <1$.

The upstream costs are the sum of the upstream treatment costs, $A(X,n, e)$ with ${A}_{X}>0$, ${A}_{XX}>0, {{ A}_{n}>0, {A}_{nn}\ge 0 \,\text{and}\, A}_{e}<0$ and upstream disutility costs (nonmonetary costs), $E(e)$, that follow from cost-containment efforts (time and energy invested into seeking cost effective solutions), where $E$ is a convexly increasing with $e$, ${E}_{e}>0$ and ${E}_{ee}>0$. Midstream costs is the sum of treatment costs, $B\left(Y,X,n, f\right),$ where ${B}_{Y}>0$, ${B}_{YY}>0$, ${B}_{n}>0, {{B}_{nn}\ge 0,B}_{X}<0,\text{ and} \,{B}_{f}<0,$ and disutility costs $F(f)$, where ${F}_{f}>0$ and ${F}_{ff}>0$. Note that upstream quality, *X*, enters the midstream treatment cost function in a negative way, ${B}_{X}<0$, hence higher upstream quality lowers midstream treatment costs (midstream externality).^16^ Finally, downstream costs is the sum of the treatment costs, $C\left(Z,Y,n, g\right)$, where ${C}_{Z}>0$, ${C}_{ZZ}>0$, ${C}_{n}>0, {{C}_{nn}\ge 0, C}_{Y}<0$, and ${C}_{g}<0$, and the disutility costs $G\left(g\right)$, where ${G}_{g}>0$ and ${G}_{gg}>0$. The presence of *Y* in $C\left(Z,Y,n, g\right)$ also introduces an externality, and since ${C}_{Y}<0,$ i.e., higher midstream quality reduces downstream costs (downstream externality).^17^,^18^ In order to simplify our forthcoming discussion, we also assume that the marginal treatment cost reduction that arises from inserting more cost-containment effort is independent of the quality levels provided and the number of patients served by all three providers, i.e. ${A}_{eX}={A}_{en}={B}_{fX}={B}_{fY}={B}_{fn}={C}_{gZ}={C}_{gY}={C}_{gn}=0$.

As concerning the benefit and cost functions, we do not impose further restrictions on the relationships between the qualities and between the qualities and the number of clients.In the same way, the marginal downstream treatment cost might decrease or increase as midstream quality increases i.e. $${C}_{YZ}\le \left(>\right)0).$$ The cross derivatives of the treatment cost functions with respect to the number of clients and the quality provided at each stage of the chain ($${A}_{Xn}$$, $${B}_{Yn},{C}_{zn})$$, can be positive (cost complementarity) or negative (cost substitutability), where a positive sign might follow if a certain quality level is more costly to achieve, the higher the number of included clients (crowding effects), while a negative sign might appear if there are learning-by-doing effects. Now let *u* $$,$$ *m* and *d* denote the sponsor transfers to the upstream, the midstream, and the downstream provider, respectively, where $$T=u+m+d$$ ($$T$$ is the total transfer).

The upstream objective function is now defined by:

1 $$\begin{array}{cc}U\left(X,Y,n\right)=\alpha V\left(X,Y,Z,n\right)-E\left(e\right)-A\left(X,n,e\right)+u&{where }\,0<\alpha \le 1\end{array}.$$

The midstream objective function follows from

2 $$\begin{array}{cc}M\left(X,Y,Z,n\right)=\beta V\left(X,Y,Z,n\right)-F\left(f\right)-B\left(Y,X,n,f\right)+m&{where }\,0<\beta \le 1.\end{array}$$

Finally, the downstream objective function becomes:

3 $$\begin{array}{cc}D\left(X,Y,Z,n\right)=\gamma V\left(X,Y,Z,n\right)-G\left(g\right)-C\left(Z,Y,n,g\right)+d&{where }\,0<\gamma \le 1\end{array}.$$

We suppose that the sponsor maximizes a social welfare function, *W*, and since the sum of the sponsor payments to the providers, *T*, is raised by distortive taxation, we introduce $\lambda$ as a measure of the marginal funding cost. Given this, the welfare function can be expressed as follows; $W=V\left(X,Y,Z,n\right)-\left(1+\lambda \right)T$ where $\lambda \ge 0$. By inserting for *T,* assuming that all providers receive compensations that (at least) cover disutilites and treatment costs, i.e. $u\ge E+A,$ $m\ge F+B$ and $d\ge G+C$, the optimal social welfare level follows from maximizing;

4 $$W=V\left(X,Y,Z,n\right)-\left(1+\lambda \right)\left\{ A\left(X,n, e\right)+ E\left(e\right)+B\left(Y,X,n, f\right)+F\left(f\right)+C\left(Z,Y,n, g\right)+G\left(g\right)\right\}$$

with regard to client group size (*n*), the qualities (*X*, *Y* and *Z*), and cost-containment efforts ($e$*, f* and *g),* where $({X}^{W},{Y}^{W},{Z}^{W},{n}^{W},{e}^{W},{f}^{W},{g}^{W})$ defines the optimal values.

The welfare-optimal condition for client group size becomes;

5 $${V}_{n}\left({X}^{W},{Y}^{W},{Z}^{W},{n}^{W}\right)=\left(1+\lambda \right)[{A}_{n}({X}^{W},{{n}^{W},{e}^{W})+B}_{n}\left({Y}^{W},{X}^{W},{n}^{W},{f}^{W}\right){+C}_{n}\left({Z}^{W},{Y}^{W},{n}^{W},{g}^{W}\right)]$$

From (5) it follows that the welfare-optimal group size is determined by equality between the marginal benefit from including an additional client and the sum of the marginal treatment costs, adjusted for the funding cost, across all three chain members. The welfare-optimal conditions for the qualities are as follows^19^:

6 $${V}_{X}\left({X}^{W},{Y}^{W},{Z}^{W},{n}^{W}\right)=\left(1+\lambda \right)[{A}_{X}({X}^{W},{n}^{W},{e}^{W} {)+B}_{X}\left({Y}^{W},{X}^{W},{n}^{W},{f}^{W}\right)]$$

7 $${V}_{Y}({X}^{W},{Y}^{W},{Z}^{W},{n}^{W})=\left(1+\lambda \right)[{B}_{Y}({Y}^{W},{{X}^{W},{n}^{W},{f}^{W})+C}_{Y}\left({Z}^{W},{Y}^{W},{n}^{W},{g}^{W}\right)]$$

8 $${V}_{Z}({X}^{W},{Y}^{W},{Z}^{W},{n}^{W})=\left(1+\lambda \right){C}_{Z}\left({Z}^{W},{Y}^{W},{n}^{W},{g}^{W}\right)$$

From (6) it follows that the marginal social benefit from higher upstream quality is to equal the sum, adjusted for the funding cost, of the marginal upstream treatment cost and the marginal midstream treatment cost reduction. The condition for midstream quality (see 7) parallels (6) since balancing the marginal social benefit with the sum of “own” marginal treatment cost and the treatment cost reduction that is experienced by the successor (the downstream provider). Finally, since downstream quality per se does not cause any externalities, the social marginal treatment cost is equal to the marginal treatment cost of the downstream provider (see 8). The first order conditions for the three cost-containment efforts are as follows:

9 $$-{A}_{e}\left({X}^{W},{n}^{W},{e}^{W}\right) = {E}_{e}({e}^{W})$$

10 $$-{B}_{f}{(Y}^{W},{X}^{W},{n}^{W},{f}^{W})={F}_{f}({f}^{W})$$

11 $$-{C}_{g}({Z}^{W},{Y}^{W},{n}^{W},{g}^{W})={G}_{g}({g}^{W})$$

All three conditions (9–11) are described by the equality between the marginal treatment cost reduction and the marginal increase in the disutility of effort.^20^

## Prospective contracts

In this section, we are concerned with pure prospective pay and how such contracts perform when it comes to coordination, and we take a positive approach, in the sense that we study how simple prospective payment rules, observed in practice, will perform. The sponsor reimburses each of the three providers according to a combination of fixed prices per client (activity-based financing) and fixed budgets. In welfare states, social care providers, rehabilitation institutions and long-term care providers (nursing homes) are typically publicly owned and reimbursed by global budgets. Furthermore, prospective payments, in terms of fixed prices, have become increasingly important for health care providers. An example of fixed prices are payments that are contingent upon the number of treated patients within a given diagnosis group (diagnosis-related groups).

The sponsor first announces the following three payment schemes; $u=u\left(n\right)={\mu }_{0}+{\mu }_{1}n,$ $m=m\left(n\right)={\eta }_{0}+{\eta }_{1}n,$ and, $d=d\left(n\right)={\delta }_{0}+{\delta }_{1}n,$ where ${\mu }_{0}$, ${\eta }_{0}$ and ${\delta }_{0}$ are lump sum payments. Since the group size is determined by the upstream provider at the first stage of the game, the transfers to the midstream and downstream provider become fixed budgets.^21^ The upstream provider determines $e$, *n* and *X*, the midstream provider determines *Y* and *f*, for a given *n*, and finally the downstream provider determines $Z\text{ and }g$ for a given *n*. The model is solved by backward induction.

From maximizing (3) w.r.t. *Z* and $g$, for given values of $(X,Y,n)$, we arrive at the following conditions for the downstream provider:

12a $$\gamma {V}_{Z}={C}_{Z}$$

and

12b $$-{C}_{g}={G}_{g}$$

Comparing (12a) with (8), it follows that the condition for optimal downstream quality deviates from the corresponding welfare-optimal condition because of the downstream provider being an imperfect agent ($\gamma <1$) and because the funding cost is ignored. From (12b), a well-known conclusion from the literature is confirmed, saying that prospective pay provides adequate cost-containment incentives (the downstream provider is internally efficient).

In the following, we characterize the downstream response function, $Z=z(Y,X,n)$, defined by the optimality conditions in (12ab). As seen from Appendix 1, the signs of ${z}_{X},{z}_{Y}$ and ${z}_{n}$ could be both positive, zero and negative.

At stage 2, the midstream provider maximizes (2) with respect to *Y* and *f,* for given values of $(X,n)$, which yields:

13a $${\beta (V}_{Y}+{V}_{Z}{z}_{Y})={B}_{Y}$$

and

13b $$-{B}_{f}={F}_{f}$$

By comparing (13b) with (10), it follows that the midstream provider faces an incentive to behave internally efficient. Besides imperfect agency and the ignorance of the funding cost, (13a) also differs from the welfare-optimal condition (see 7), since the midstream provider does not internalize the cost externality (${C}_{Y}<0$). Furthermore, (13a) differs from the welfare-optimal condition due to the presence of a strategic effect that arises from the downstream response function (${z}_{Y}\ne 0$). Using (12a), the condition in (13a) can be rewritten as follows;

14 $${\beta V}_{Y}= {B}_{Y}-{\beta V}_{Z}{z}_{Y}{=B}_{Y}-\frac{\beta }{\gamma }{C}_{Z}{z}_{Y}$$

The last term of the right-hand side of (14) represents the strategic effect. For a positive (negative) response, i.e., the qualities are strategical complements (strategical substitutes), the midstream provider chooses a higher (lower) quality level, *Y,* to induce the downstream provider to increase the level of $Z$ (relatively to the case of strategic independent efforts; ${z}_{Y}=0$). Furthermore, the significance of the strategic effect, *ceteris paribus*, increases with a higher $\beta$ and a lower $\gamma$. Consequently, the more the midstream provider values client benefits, and the less the downstream provider values the same benefits, the stronger is the strategic incentive.

The midstream reaction function, $Y=y(X,n)$, is defined by (13a) and (13b). In Appendix 1, the signs of ${y}_{X}$ and ${y}_{n}$ are discussed in more detail (can be both positive, zero and negative).

At stage 1, the upstream provider maximizes (1) with respect to *X*, $e$ and *n*. The optimality conditions for *X* and *e* become as follows:

15a $$\alpha \left[{V}_{X}+{V}_{Y}{y}_{X}+{V}_{Z}\left({z}_{Y}{y}_{X}+{z}_{X}\right)\right]={A}_{X}$$

and

15b $$-{A}_{e}={E}_{e}$$

By comparing (15b) with (9), it follows that the upstream provider has an incentive to behave internally efficient. Besides preference misalignment problems (imperfect agency and the ignorance of the funding cost), the upstream quality condition deviates from the relevant welfare-optimal condition (see 6) since ignoring the cost externality (${B}_{X}<0$) and because of the presence of two strategic effects, where one derives from the midstream response function, ${y}_{X}\ne 0$, while the other derives from the downstream response function, ${z}_{X}\ne 0$. The condition in (15a) can be rewritten as follows:

15c $$\alpha {V}_{X}={A}_{X}-\alpha \left[{V}_{Y}{y}_{X}+{V}_{Z}\left({z}_{Y}{y}_{X}+{z}_{X}\right)\right]= {A}_{X}-\frac{\alpha }{\beta }{B}_{Y}{y}_{X}-\frac{\alpha }{\gamma }{C}_{Z}{z}_{X}$$

The last two terms on the right-hand side of (15c) represent the strategic effects. For ${y}_{X}>(<)0$, higher (lower) upstream quality induces the midstream provider to increase (decrease) its quality. *Ceteris paribus*, this effect increases with a higher $\alpha$ and a lower *β.* For ${z}_{X}>\left(<\right)0$, higher (lower) upstream quality induces the downstream provider to increase (decrease) its quality. This effect increases with a higher $\alpha$ and a lower γ, *ceteris paribus*. The optimality condition for client group size, *n*, is:

15d $$\alpha \left[{V}_{n}+{V}_{Y}{y}_{n}+{V}_{Z}\left({z}_{Y}{y}_{n}+{z}_{n}\right)\right]+{\mu }_{1}={A}_{n}$$

Besides the preference misalignment problems, the optimal upstream condition for group size deviates from the relevant welfare-optimal condition since the upstream provider ignores the two group size externalities (${B}_{n}>0$ and ${C}_{n}>0$). In addition, two strategic effects induce deviations from the welfare-optimal condition (${y}_{n}\ne 0$ and ${z}_{n}\ne 0).$

The expression for the optimal group size can be rewritten as follows:

15e $$\alpha {V}_{n}+{\mu }_{1}={A}_{n}-\alpha \left[{V}_{Y}{y}_{n}+{V}_{Z}\left({z}_{Y}{y}_{n}+{z}_{n}\right)\right]= {A}_{n}-\frac{\alpha }{\beta }{B}_{Y}{y}_{n}-\frac{\alpha }{\gamma }{C}_{Z}{z}_{n}$$

From (15e) it follows that the two strategic effects depend on the altruistic preferences. First, both effects, *ceteris paribus,* become more significant with a higher degree of upstream altruism. Second, the effect associated with midstream quality decreases with a higher degree of midstream altruism, and third, the effect associated with downstream quality, *ceteris paribus,* decreases with a higher degree of downstream altruism. It also follows from (15e) that a higher price, ${\mu }_{1}$, *ceteris paribus*, stimulates the upstream provider to include more clients.

To sum up, for the quality variables and group size, the upstream optimality conditions differ from the relevant welfare-optimal conditions, due to imperfect agency, the ignorance of the funding cost, the non-internalization of cost externalities and the presence of a series of strategic effects.^22^ In sum these problems induce a suboptimal group size at the same time as the care pathway is characterized by suboptimal quality levels at every stage of the production chain.^23^ Additionally, it should be remarked that for these prospective contracts to be accepted by the providers, the transfers must satisfy $u\ge A+E$*,* $m\ge B+F$ and $d\ge C+G$. In cases where the contracts imply transfers above the necessary levels, i.e., $u>A+E$*,* $m>B+F$ and/or $d>C+G$, there is a lack of efficiency in the funding as long as $\uplambda>0$. Generally, the welfare loss related to the extra funding is defined by $\lambda \{\left[u-\left(A+E\right)\right]+\left[m-\left(B+F\right)\right]+\left[d-\left(C+G\right)\right]$.

The above conclusions prevail if the upstream reimbursement rule is assumed to be independent of the number of clients, i.e., ${\mu }_{1}=0.$ In this case, the contracts become as follows; $u={\mu }_{0}, m={\eta }_{0}$ and $d={\delta }_{0}$ (fixed budgets) and the optimality conditions coincide with those derived when a fixed price was included, with one exception, ${\mu }_{1}=0$ in (15e). It should be noted that the upstream provider is confronted with two types of strategic incentives with respect to quality (see 15c; ${y}_{X}\ne 0\text{ and }{z}_{X}\ne 0)$ and two types of strategic incentives with respect to group size (see 15e; ${y}_{n}\ne 0$ and ${z}_{n}\ne 0)$. Hence, the strategic incentives for any provider are not limited to the next provider in the production chain but to all subsequent providers.^24^ Our model simplifies if the group size is determined by the sponsor since reducing the number of externalities and strategic effects, however, the main mechanisms of the model prevail.^25^

Consider now the case where the degree of agency, for a given provider, is in accordance with the preferences of the sponsor (perfectly adjusted with the funding costs), in the following denoted as “perfect agency**”.** If all three providers are perfect agents; $\frac{1}{1+\lambda }=\gamma =\alpha =\beta$, the welfare-optimal solution becomes attainable under pure prospective pay only if; (i) the externalities are absent, i.e. ${B}_{X}={C}_{Y}={B}_{n}={C}_{n}=0$, and, (ii) there are no incentives to behave strategically, i.e. ${y}_{X}={y}_{n}={z}_{Y}={z}_{X}={z}_{n}=0$. If some externalities and/or strategical effects are present, the welfare-optimal solution becomes unattainable despite the providers being perfect agents. For instance, if more clients lead to higher midstream and downstream costs, i.e., ${B}_{n}>0$ and ${C}_{n}>0$, there is an upstream incentive for including too many clients in the production chain and this is reinforced for a positive price per client, i.e., ${\mu }_{1}>0$*.*

### Result 1

*For a pure prospective scheme, all providers face adequate cost containment incentives. However, each of the following factors contribute to a suboptimal group size and suboptimal quality levels at all stages of the production chain; (i) the providers ignore funding costs, i.e.*$\lambda>0$*, (ii) one or more providers are imperfect agents, (iii) the presence of externalities that originate from the midstream treatment cost function (*${B}_{X}<and{B}_{n}>0$*) and the downstream treatment cost function *${(C}_{Y}<0 and {C}_{n}>0),$* and, (iv) the presence of strategic incentives (*${y}_{X}\ne 0$*,*${y}_{n}\ne 0{z}_{X}\ne 0$*, *${z}_{n}\ne 0$* and* ${z}_{Y}\ne 0).$

## Cost-based contracts

We now consider the case where both group size and ex-post treatment costs are contractible. Such information implies that the reimbursement rule for each provider in principle can be made contingent upon the treatment costs of all production chain partners. In the following, only the treatment costs of subsequent partners are considered since predecessor costs are of no value to the sponsor. The following linear contracts will be discussed;

16a $$u=u\left(A,B,C,n\right)={u}_{0}+{u}_{1}A+{u}_{2}B+{u}_{3}C+{u}_{4}n$$

16b $$m=m\left(B,C\right) ={m}_{0}+{m}_{1}B+{m}_{2}C$$

16c $$d=d\left(C\right) ={d}_{0}+{d}_{1}C$$

where ${u}_{i},{m}_{j}$ og ${d}_{k}$ are constants, $i=\text{0,1},\text{2,3},4$, $j=\text{0,1},2$ and *k* = *0,1* and where $u\left(A,B,C,n\right)$ denotes the transfer from the sponsor to the upstream provider, $m\left(B,C\right)$ the transfer to the midstream provider and $d\left(C\right)$ the transfer to the downstream provider. The parameters ${u}_{1, } {m}_{1}$ and ${d}_{1}$ represent shares based on “own” costs, in the following denoted “cost-sharing” parameters, while ${u}_{2, } {u}_{3} \text{and} {m}_{2}$ are shares based upon the costs of the subsequent providers, in the following denoted “integrated” parameters. Furthermore, ${u}_{4}$ is the fixed payment per client, while ${u}_{0},$ ${m}_{0}$ and ${d}_{0}$ are lump sum payments (fixed budgets).

The derivations of the second-best contracts will be complex since the sponsor, in total, must decide on ten different parameters. For this reason, to simplify our discussions, we restrict our analysis to the identification of the parameter values that make the optimal quality conditions to coincide with the relevant welfare-optimal conditions (see 6–8) in combination with a discussion of what the implications will be for cost-containment. As before the game is solved by backward induction, where the sponsor first announces the contract terms (16abc), thereafter the upstream provider determines *n,* $X$ and $e$, followed by the midstream provider deciding on *Y* and $f,$ and finally, the downstream provider determines $Z$ and $g.$

The optimality conditions for the downstream provider, with respect to *Z* and *g,* are as follows:

17a $${D}_{Z}=\gamma {V}_{Z}+({d}_{1}^{*}-1){C}_{Z}=0$$

17b $$-\left(1-{d}_{1}^{*}\right){C}_{g}={G}_{g}$$

Now by comparing (17a), with the relevant welfare-optimal condition (8), it follows that the parameter value that makes the two conditions to coincide is;

17c $${d}_{1}^{*}=1-\left(1+\lambda \right)\gamma$$

Furthermore, since the downstream provider must be offered an acceptable contract (i.e. ${d}^{*}\ge C+G$), we get that ${d}_{0}\ge \left(1-{d}_{1}^{*}\right)C+G$ and if ${d}_{0}^{*}$ is defined as the lowest possible lump-sum payment, we have that ${d}_{0}^{*}=G+\left(1+\lambda \right)\gamma C.$ From (17c), we observe that the cost-sharing parameter, ${d}_{1}^{*},$ concerned with downstream preference alignment, is to equal the deviation between the sponsor’s valuation of benefits and the downstream valuation of the same benefits adjusted for the funding costs (“adjusted non-internalized benefits”).

For the case where $\frac{1}{1+\lambda }=\gamma$ (“perfect downstream agency”) we get ${d}_{1}^{*}$ = 0 and ${d}_{0}^{*}=C+G,$ thus the downstream contract becomes a pure prospective one and the condition for internal efficiency holds. For $\frac{1}{1+\lambda }>\gamma$ (too low downstream altruism), the payment to the downstream provider, *ceteris paribus*, increases with higher downstream costs (${d}_{1}^{*}>0$ is termed as positive cost-sharing**)**, but at a lower rate than the full costs since $0<{d}_{1}^{*}<1$. Also in this case, the contract includes a prospective element which (at least) covers the remaining part of the treatment costs and the provider’s disutility, i.e., ${d}_{0}^{*}<C+G$. Given positive cost-sharing (${d}_{1}^{*}>0$), we observe from (17b), that the downstream provider now inserts less effort into cost-containment compared to what is preferred from a welfare point of view (see 11). For $\frac{1}{1+\lambda }<\gamma$ (too much downstream altruism), the payment to the downstream provider, *ceteris paribus*, decreases with higher downstream costs (${d}_{1}^{*}<0$ is termed as negative cost-sharing). In this case, the provider becomes fully responsible for own costs, but, in addition, this provider must pay the sponsor a fee that is proportional with own costs. In the case of negative cost-sharing (${d}_{1}^{*}<0$), it now follows from (17b), relative to the welfare-optimal level (see 11), that the downstream provider exerts too much effort into cost-containment. Moreover, in this case the minimal lump-sum payment must be higher than the sum of treatment costs and the provider’s disutility, i.e., ${d}_{0}^{*}>C+G$.

The optimality conditions for the midstream provider with respect to *Y* and *f*, are as follows^26^;

18a $${M}_{Y}={\beta (V}_{Y}+{V}_{Z}{z}_{Y})+\left({m}_{1}^{*}-1\right){B}_{Y}+{m}_{2}^{*}({C}_{Z}{z}_{Y}+{C}_{Y})=0$$

and

18b $$-\left(1-{m}_{1}^{*}\right){B}_{f}={F}_{f}$$

where ${z}_{Y}$ is the derivates of the upstream provider’s response function, implicitly defined by (17ab), for details on the characteristics of this function, see Appendix 2. Then, by comparing (18a) with the welfare optimal condition for midstream quality (see 7), it follows that the two conditions coincide for the following three parameter values:

18c $${m}_{1}^{*}=1-\left(1+\lambda \right)\beta$$

18d $${m}_{2}^{*}=-\left(1+\lambda \right)\beta$$

and,

18e $${m}_{0}\ge \left(1-{m}_{1}^{*}\right)B+F-{m}_{2}^{*}C$$

The parameters of (18c) and (18d) follows from comparing with (7), while (18e) follows from the midstream participation constraint, i.e. ${m}^{*}\ge B+F$. Moreover, let ${m}_{0}^{*}$ be the minimum value that satisfies (18c), i.e. ${m}_{0}^{*}=\left(1-{m}_{1}^{*}\right)B+F-{m}_{2}^{*}C$.

As was the case for the downstream contract, the cost-sharing parameter for the midstream provider (see 18e) is concerned with preference alignment where ${m}_{1}^{*}$ now is to equal the deviation between the sponsor’s valuation of client benefits and the midstream valuation of the same benefits adjusted for the funding costs (“adjusted non-internalized benefits”). In addition, from (18d), it follows that an integrated parameter, ${m}_{2}^{*}$, now is part of the contract and this parameter determines the weight given to the treatment costs of the subsequent provider (the downstream provider). The integrated parameter, ${m}_{2}^{*}=-\left(1+\lambda \right)\beta <0,$ is always negative meaning that higher downstream treatment costs impact the midstream provider in a negative way and the significance of this “responsibility” is proportional to the degree of “adjusted internalized benefits” that can be both lower and higher than -1. This parameter, however, does not imply any cost-sharing, since a higher midstream “cost responsibility” does not reduce the responsibility of the downstream provider for “own” costs, hence this parameter acts as a penalty, and it is integrated since being contingent upon the treatment costs of the production chain followers (“an integrated penalty”). The integrated penalty addresses the following two coordination problems; (i) the presence of the cost externality imposed onto the downstream provider, and (ii) the presence of the strategic incentive held by the midstream provider.

Given perfect midstream agency, $\frac{1}{1+\lambda }=\beta$, it follows from comparing (18b) and (12) that the contract becomes a mixed one consisting of prospective pay and an integrated penalty that is equal to downstream costs (${m}_{1}^{*}=0$ and ${m}_{2}^{*}=-1$). This implies, due to the absence of cost-sharing, that the welfare-optimal solution will be realized.^27^ Given a high degree of midstream altruism, $\frac{1}{1+\lambda }<\beta$, the contract becomes a mixed one consisting of the following three elements: prospective pay, an integrated penalty (${m}_{2}^{*}>-1$), and negative cost-sharing. In this case, the midstream provider will put too much effort into cost-containment. For a low degree of midstream altruism, $\frac{1}{1+\lambda }>\beta$, the contract consists of prospective pay, an integrated penalty (${m}_{2}^{*}<-1$), and positive cost-sharing. In this case, the midstream provider will insert too little effort into cost- containment.

The condition for the quality chosen by the upstream provider, *X*, and the condition for upstream effort, *E*, are^28^:

19a $${U}_{X}=\alpha \left[{V}_{X}+{V}_{Y}{y}_{X}+{V}_{Z}\left({z}_{Y}{y}_{X}+{z}_{X}\right)\right]+\left({u}_{1}^{*}-1\right){A}_{X}+{u}_{2}^{*}{({B}_{Y}{y}_{X}+B}_{X})+{u}_{3}^{*}\left[{C}_{Z}{(z}_{Y}{y}_{X}+{z}_{X}\right)+{C}_{Y}{y}_{X}]=0$$

19b $$-\left(1-{u}_{1}^{*}\right){A}_{e}={E}_{e}$$

Moreover, the condition for client group size becomes:

19c $${U}_{n}=\alpha \left[{V}_{n}+{V}_{Y}{y}_{n}+{V}_{Z}\left({z}_{Y}{y}_{n}+{z}_{n}\right)\right]+\left({u}_{1}^{*}-1\right){A}_{n}+{u}_{2}^{*}{({B}_{Y}{y}_{n}+B}_{n})+{u}_{3}^{*}\left[{C}_{Z}{(z}_{Y}{y}_{n}+{z}_{n}\right)+{C}_{Y}{y}_{n}+{C}_{n}] +{u}_{4}^{*} =0$$

For the upstream provider, the parameter values are;

19d $${u}_{1}^{*}=1-\left(1+\lambda \right)\alpha$$

19e $${u}_{2}^{*}={u}_{3}^{*}=-\left(1+\lambda \right)\alpha$$

19f $${u}_{4}^{*}=0$$

and

19g $${u}_{0}\ge \left(1-{u}_{1}^{*}\right)A+E-{u}_{2}^{*}\left(B+C\right)$$

The values defining ${u}_{1}^{*}$, ${u}_{2}^{*}$, ${u}_{3}^{*}$ and ${u}_{4}^{*}$ follow directly from comparing (19a) and (19b) with Eqs. (5) and (6), while (19 g) follows from the upstream participation constraint, i.e., ${u}^{*}\ge A+E$. Moreover, let ${u}_{0}^{*}$ be the minimum value of (19 g) that satisfies this condition, i.e., ${u}_{0}^{*}=\left(1-{u}_{1}^{*}\right)A+E-{u}_{2}^{*}\left(B+C\right)$.

Again the “cost-sharing” parameter, ${u}_{1}^{*}=1-\left(1+\lambda \right)\alpha$, is concerned with preference alignment. Furthermore, there are now two “integrated” parameters, ${u}_{2}^{*}$ and ${u}_{3}^{*},$ both addressing coordination imperfections that lie with the decision-making of the upstream provider (the cost externalities and the strategic incentives). Furthermore, since ${u}_{4}^{*}=0$ (see 19f), the sponsor need not to include a payment per client as part of the contract to align the incentives for group size, since this problem is already taken care of by the two “integrated” parameters.

For perfect upstream agency, $$\frac{1}{1+\lambda }=\alpha$$, $${u}_{1}^{*}$$ equals zero and $${u}_{2}^{*}={u}_{3}^{*}=-1$$. Thus, we have a situation with no cost-sharing, one integrated penalty that is to equal midstream treatment costs and one integrated penalty that is to equal downstream treatment costs (complete internalization). Since cost-sharing is not part of the contract, the midstream condition for internal efficiency holds. For $$\frac{1}{1+\lambda }<$$ $$\alpha$$ (a high degree of upstream altruism), the contract consists of prospective pay, integrated penalties that are higher than -1 ($${u}_{2}^{*}={u}_{3}^{*}>-1)$$, and negative cost-sharing ($${u}_{1}^{*}<0)$$ inducing too much upstream cost-containment effort. For $$\frac{1}{1+\lambda }<$$ $$\alpha$$ (a low degree of upstream altruism), the contract consists prospective pay, integrated penalties that are lower than -1 ($${u}_{2}^{*}={u}_{3}^{*}<-1)$$, and positive cost-sharing ($${u}_{1}^{*}>0)$$ inducing too little upstream cost-containment effort.

The above discussions show that the contracts are typically mixed contracts that combine prospective pay with positive or negative cost-sharing and integrated penalties. Furthermore, the contracts differ across chain members. To illustrate this, consider the case where the upstream provider is non-altruistic (α → 0) and where the two remaining providers are assumed to be perfect agents; $\frac{1}{1+\lambda }=\beta =\gamma .$ In this case, the upstream contract becomes a cost-sharing contract (${u}_{0}^{*}\to E, {u}_{1}^{*}\to 1,{u}_{2}^{*}=0 \text{and}{ \mu }_{3}^{*} =0),$ the midstream contract becomes one that combines prospective pay with one integrated penalty (${m}_{0}^{*}=B+C+F \text{and }{m}_{2}^{*}=-1$), while the downstream contract becomes a pure prospective contract ${(d}_{0}^{*}=C+G,{d}_{1}^{*}=0)$. As concerning cost-containment, the upstream provider has no such incentives (${u}_{1}^{*}\to 1)$, while the remaining two providers $({m}_{1}^{*}={d}_{1}^{*}=0$) are confronted with adequate cost-containment incentives. To conclude, the cost-based contracts eliminate preference misalignments and the coordination problems among the production chain partners (externalities and strategic effects) that are present when practicing pure prospective contracts, as stated in Result 1 above. However, when cost-sharing becomes necessary to adjust for preference misalignments for one or more chain partners, internal inefficiencies are introduced meaning that the sponsor, when determining the second-best contracts, must balance concerns for quality with internal inefficiency concerns.

### Result 2

*In s*e*quential production chains, the distortions that follow from preference misalignments and poor coordination can be eliminated by using contracts that are contingent upon ex-post treatment costs. These contracts will be mixed ones that include prospective pay, a cost-sharing element (positive or negative), and integrated penalties. The structure of the contracts differs across the partners depending on their position in the production chain. The first best solution is attained only in the special case where all partners are perfect agents,* $\frac{1}{1+\lambda }=\alpha =\beta =\gamma .$

The various contracts that are derived in this section are summarized in a Table with comments in Appendix 3. An interesting observation is that all contracts need not change if cost externalities are absent. In such a case, the treatment costs of the midstream provider and the downstream provider, respectively, become as follows; *B(Y,n,f)* and *C(Z,n,d).* Now,${u}_{3}^{*}$ changes and becomes equal zero while ${u}_{2}^{*}$ and ${m}_{2}^{*}$ remain unchanged; ${u}_{2}^{*}=-\left(1+\lambda \right)\alpha$ and ${m}_{2}^{*}=\left(1+\lambda \right)\beta$. These findings follow since, in addition to ensuring the welfare-optimal group size, ${u}_{2}$ and ${m}_{2}$ serve two purposes while ${u}_{3}^{*}$ serves one purpose. ${u}_{2}$ ensures that (i) the upstream provider internalizes the effect own decision making has on midstream treatment costs, and (ii) the strategic incentives faced by the upstream provider towards the midstream provider are alleviated. ${m}_{2}$ ensures that (i) the midstream provider internalizes the effect own decision making has on downstream treatment costs, and (ii) the strategic incentives faced by the midstream provider towards the downstream provider are alleviated. ${u}_{3}$, on the other hand, only alleviate the strategic incentives faced by the upstream provider towards the downstream provider since no externality arises from the downstream quality decision.

As for pure prospective contracts, the providers’ disutilities are supposed to be unobservable, meaning that the sponsor might transfer more funds than necessary. Given a funding cost, i.e., $\lambda>0$, this implies that society experiences an extra welfare loss. Using the definitions above, this welfare loss is equal to $\lambda {[(d}_{0}-{d}_{0}^{*})+{[(m}_{0}-{m}_{0}^{*})+{(u}_{0}-{u}_{0}^{*})]$. However, unlike the case of pure prospective pay, the sponsor can now use ex-post treatment costs to estimate part of the providers’ funding requirements (*A*, *B* and *C*), despite of the disutilities being unobservable, i.e., the values of (*E*, *F* and *G*). This implies that it might be easier for the sponsor to calculate what the necessary transfers are, thus limiting the efficiency loss compared to the prospective case where ex post treatment costs are not part of the contract.

## Discussion

As the number of vulnerable clients in need of services from several providers is increasing, an effective organization of sequential care chains becomes imperative. We have shown that pure prospective pay in terms of a fixed budget, a fixed price, or a combination of the two, gives rise to several inefficiencies. First, because prospective contracts are not able to address the problems that follow from preference misalignments and coordination disincentives. Second, prospective contracts create an upstream incentive for being too generous when it comes to the inclusion of clients. On the other hand, prospective pay ensures the welfare-optimal levels of cost containment activities (internal efficiency).

We also show that the introduction of contracts that utilize ex-post treatment cost information has the potential for improving the situation relative to pure prospective contracts. This conclusion follows since cost-based contracts enable the sponsor to apply both cost-sharing and integrated penalties to steer the allocation of quality among chain partners. The cost-sharing elements of the contracts, being positive or negative, enable the sponsor to align the provider preferences with the preferences of society (the sponsor). Positive cost-sharing refers to the case where the sponsor partly subsidizes the costs of a given provider, and this instrument becomes desirable when the degree of provider agency is too low to induce the quality level being preferred by society. Negative cost-sharing refers to a situation where the payment decreases with higher treatment costs, meaning that the provider is fully responsible for own costs, but, in addition, must pay the sponsor a fee that that is proportional to own treatment costs.

The integrated penalties, being contingent upon the treatment costs of subsequent chain partners. eliminate coordination problems, and these penalties can be both higher and lower than the actual treatment costs of the production chain followers. The use of the integrated penalties implies that a given provider will benefit from the cost-containment investments undertaken by subsequent chain partners, however, the provider in question will not be able to act on such incentives since being under the control of the chain partners. To the extent cost-sharing becomes necessary for aligning provider preferences, the incentive for investing into cost containment activities becomes lower, implying that the social benefits that arise from cost-based contracts must be balanced against internal inefficiency concerns**.**

Ellis and McGuire [24] ignore cost-containment issues when studying a single altruistic provider that decides on non-contractible quality. Given perfect agency, the welfare-optimal solution in this case can be reached under pure prospective pay. For imperfect agency, the welfare-optimal solution follows from a mixed contract that contains a prospective element in combination with cost-sharing. In our analysis, when ignoring cost containment issues ($$e=f=g=0$$ and $${A}_{e}={B}_{f}={C}_{g}=0)$$, we find, in contrast to Ellis and McGuire [24], under pure prospective pay and all providers being perfect agents ($$\alpha =\beta =\gamma =\frac{1}{1+\lambda }$$), that the welfare-optimal solution is unattainable. The difference in conclusions lies with the interdependencies that exist between the sequential chain partners (cost externalities and strategic incentives) in a sequential framework. However, by introducing integrated funding (integrated penalties), welfare-optimality can be reached since cost-sharing is not in demand given perfect agency. If some, or all, production chain partners are imperfect agents, the welfare-optimal solution becomes unattainable since now cost-sharing (positive or negative) is needed for ensuring that the provider preferences become perfectly aligned with the social preferences.

Chalkley and Malcomson [17] extends the framework of Ellis and McGuire by also considering cost containing incentives, and find, for perfect agency, that the welfare-optimal contract is a pure prospective one. In our sequential framework, however, the welfare-optimal solution will typically be unattainable under pure prospective pay due to the presence of the coordination disincentives. For an imperfect agent, Chalkley and Malcomson [17] arrive at a conclusion like ours in the sense that the welfare-optimal solution becomes unattainable. In both works, cost-sharing becomes necessary for aligning the provider preferences with the social preferences. Now, the sponsor must balance the quality benefits that follow from cost-sharing with the inefficiency losses that follow from cost-sharing.^29^ However, in our sequential framework, the use of cost-based contracts becomes relative more attractive relatively to pure prospective pay, if compared with a single-provider perspective. This conclusion follows because a chain perspective introduces coordination challenges that can be modified or eliminated by using integrated penalties. If fact, in situations where a sponsor chooses not to introduce cost-sharing out of internal efficiency concerns, the use of integrated penalties may still improve social welfare since eliminating the incentives for poor coordination.

Winter [89], when analyzing a moral hazard model in which agents move sequentially, finds that agents (i) should be rewarded according to their positions in the production chain, and (ii) early movers should be confronted with more low-powered incentives relatively to late movers since early movers face a higher implicit threat (are followed by a higher number of decision-makers). The first conclusion of Winter [89] is in line with our findings, however, the overall significance of the incentives directed at the early movers, relatively to those directed at the late movers, in our analysis will depend on the degree of altruism held by the various providers in combination with their position in the production chain. However, these two dimensions interact in complex ways, implying that determinate conclusions cannot be reached.

Our analysis shows that cost-based contracts contain informational requirements that go beyond the contractability of ex-post treatment costs since information about funding costs and altruistic preferences are needed as well. Estimates on the opportunity cost of public funds are available for several countries. For example, the official rates for project appraisal in the Scandinavian countries lie between 0.2 and 0.3 [60]. There is also an empirical literature on physician altruism (patient-regarding preferences) that finds that patients’ health benefits, in addition to payments, play a role for clinicians (see the refences in the introduction 1). However, evidence on variation across firms, institutions and organizations is lacking. At the individual level, altruism appears to be private information, but this is less obvious at the organizational level. Repeated interactions between sponsors and providers and the possibility of sponsors to acquire relevant information about culture and management styles (e.g., patient satisfaction studies, evaluations, and audits) suggest that some relevant information is available or can be collected at reasonable costs.^30^

The main mechanism of cost-based contracts, when it comes to providing adequate coordination incentives, is that any chain partner, to some degree, must internalize the treatment costs of the subsequent partners (integrated penalties). A natural question now becomes to what extent this principle has real-world applications. The evidence base on the effects from integrated funding includes a substantial number of primary studies concerned with both intersectoral and intra-sectoral interventions (see the references in the introduction). Despite this, it is difficult to draw valid and reliable conclusions based on this literature. The overall impression from the reviews is that the evidence is mixed where some primary studies report about positive results in terms of improved quality perceptions, increased client satisfaction and improved access to care, while the costs typically stay constant or become higher.^31^

As concerning the various interventions (applications), some are limited in the sense that the ambition is mainly to support specific projects and actions rather than promoting coordination on a more general basis and because they typically represent small shares of the total income for the involved providers [66]. More promising interventions is when two more providers or sectors are reimbursed jointly (the pooling of funds). For such initiatives some degree of financial integration is taking place since the funding streams of the participating providers are not entirely separate. Examples of such initiatives are bundled payments (a defined part of the care pathway is reimbursed by a single tariff) and population-based payments (a group of providers receives a pooled budget as compensation for being responsible for the delivery of certain services to a pre-determined population).^32^ The pooling of funds ties the providers together, but to what extent such a tying leads to an internalization of the treatment costs of chain partners remains an open question.^33^ A decisive factor in this respect is probably the principles applied for the sharing of the pooled funds among the participating providers.

A natural question is to what extent do our theoretical findings, as concerning the role of integrated penalties and the role cost sharing, as coordination mechanisms, resemble real-world applications? An example of a reform that builds upon the principle of integrated penalties, is implemented in Denmark. The structural health care reform of 2007 meant that the municipalities had to pay a share of the hospital costs each time a municipal resident was admitted to a regional hospital (municipal co-sharing). The idea was to create incentives for municipalities to increase preventive services to reduce hospital admissions. A similar Norwegian reform was implemented in 2012; however, this reform was short-lived due to political concerns about the municipalities withholding vulnerable patients to save funds. Surveys concerned with the Danish structural health care reform confirm that many municipalities responded by spending more resources on prevention, however, a systematic study from 2013 on the relationships between public health efforts and the number of admissions among the elderly (age 67 +) for four diagnostic groups, did not establish any clear links [85].

There are also real-world examples of cost-sharing being introduced as a coordinating mechanism. One example is the role of GP referral incentives. Theoretical studies on gatekeeping find that GPs that are reimbursed by fee-for -service (FFS), relatively to GPs paid by pure capitation, are less likely to refer patients to specialized care since being fully compensated (complete cost-sharing) for treating patients [1]. This finding is also consistent with empirical evidence [38]. Thus, one possible reason for the frequent use of mixed GP reimbursement schemes consisting of partial cost-sharing (FFS) in combination with capitation, could be to avoid unnecessary referrals. There are also other types of initiatives that aim to provide adequate referral incentives. For example, some GP clinics in the UK are reimbursed contingent upon meeting specific referral-reduction targets [77]. Related examples from the US include some state Medicaid programs where financial disincentives (penalties) are applied to discourage unnecessary referrals to high-cost specialty care [55].

The implementation of financial incentives is not straightforward since various conditions need to be fulfilled for such incentive schemes to become effective. Eijkenaar [23], when discussing key elements of appropriate design, refers to *what* (the type decisions that are incentivized), *how* (the structure of the incentive schemes) and *who* (the identity of those receiving the performance awards). Other literature concerned with optimal design mention pay-off formulas and awareness (see e.g. [92]). Payoff formulas refer to types of incentives (rewards or penalties), incentive structure (absolute or relative), and factors such as frequency, duration, and magnitude while awareness refers to involvement, transparency and legitimacy. It may be that the use of integrated penalties is challenging in relation to ensure legitimacy among stakeholders. The idea of letting revenues become dependent on the performance (treatment costs) of others might be perceived as being unfair, which may trigger resistance to the use of such a policy instrument. For integrated penalties to be legitimate, their objectives must be well-communicated and understood.

In our model, the upstream provider selects the group of clients that enters into the care pathway; however, other alternatives are possible. For example, for health care pathways in national health systems, the client group typically follows from inclusion criteria being defined by the sponsor.^34^ Whether the group size decision is left with the sponsor rather than the upstream provider, simplifies our analysis, but does not change our main findings. Another possibility would be to introduce a demand function for care pathways; hence client choice represents a mechanism for ensuring quality. In the literature, several contributions discuss the role of quality and cost containment incentives when quality is driven by demand rather than agency [17, 32, 62, 75]. An important conclusion is that demand-driven quality may substitute for cost-sharing in promoting quality, thus the welfare-optimal solution becomes attainable under pure prospective contracts [62]. Clients in welfare states, however, do seldom choose between care pathways because of catchment area responsibilities and capacity problems (waiting lists). In addition, the client groups in question are typically consisting of vulnerable individuals (elderly with high comorbidity, the terminally ill, patients with chronic diseases and mental illnesses, and drug abusers) that, most likely, are without the capacity and resources to make informed choices.

Quality can also be promoted by other institutions. One example is accreditation (certification) that produces much the same incentives as demand-driven quality, since the accreditation bodies, when issuing certificates, verify that certain quality standards are fulfilled. If, however, clients do not respond to the accreditation status of providers, the incentives for providers to seek accreditation are weak or absent. In such situations, mandatory accreditation may act as imperfect) substitute to demand-driven quality, in this way reducing the need for using cost-sharing contracts to promote quality.^35^

Our analysis is based on some simplifying presumptions. For instance, it might be more reasonable to assume that the marginal treatment costs are decreasing in cost-containment efforts, for instance ${A}_{eX}<0, {A}_{en}<0,{B}_{fY}<0,{B}_{fn}<0,{C}_{gZ}<0$ and ${C}_{gn}<0$. Such assumptions imply that it, at the margin, becomes more advantageous for the sponsor to choose contracts that stimulate the providers to invest in cost reducing activities. Second, we have not explicitly included any stochastic variables. For instance, it is reasonable to believe that care production is affected by unverifiable stochastic events making it difficult for the sponsor to reveal what explains the observed treatment costs. However, simplifying the model by ignoring possible stochastic variables does not influence our reasonings given risk neutral decision-makers, since now the optimal contracts need not pay concerns to risk allocation. For example, hospitals as health care providers and municipalities as social care providers, are typically large organizations that might be considered as being risk neutral. On the other hand, for providers such as primary care centers, rehabilitation centers and long-term care providers, risk is clearly an issue that must be considered. From the literature, we know that cost reimbursement represents less risk relative to prospective contracts. However, integrated penalties may expose providers too significant risks, and we find that integrated penalties become more important the higher the degree of altruism held by each provider. In this perspective, a strong concern for clients may induce optimal contracts that impose significant risks on some providers.

Third, selection problems are not considered. The literature confirms that prospective payments promote risk selection activities such as admitting higher value patients (cherry-picking) while deterring patients expected to generate financial losses (dumping). A well-known result is that cost-sharing reduces such incentives, however, it is not self-evident how the presence of integrated penalties impacts such incentives. In contrast to single-provider frameworks, a sequential perspective introduces additional challenges since the transfer of clients from one provider to another becomes important for the distribution of costs along the care pathway. Given prospective payments, there is an incentive for each provider to transfer patients at an early stage, in this way reducing own responsibility at the expense of chain partners (“quicker and sicker”). However, for contracts that involve some degree of cost-sharing, such incentives are weaker, but the same conclusion does not appear valid for integrated penalties. Fourth, in our sequential framework, downstream providers are not allowed to undertake obstructive behavior. However, such behavior, for example in terms of bed-blocking, is observed in many health care systems. Fifth, the literature on integrated care refers to some patients as being “revolving door patients” in the sense that they are switching back and forth between the same providers over a long period of time. One interpretation of such observations is that they follow from uncoordinated care. An extension of our model would be to study “revolving door patients” as well incentives for obstructive behavior.^36^

## Conclusion

Based on a simplified model, we study the welfare implications from using cost-based contracts that target providers that act in sequential production chains. The specified contracts contain parameters that represents shares of the treatment costs for the provider in question (positive or negative cost-sharing) as well as parameters that hold providers responsible for the treatment costs of their subsequent chain partners (integrated penalties). One of the main conclusions of this analysis is that proposed cost-based contracts are typically mixed contracts that address two different types of problems (inefficiencies) that occur under pure prospective pay. The first type of problem is not coordination problems but points to problems that arise because of misaligned provider preferences and these problems are addressed by introducing cost-sharing contracts. The second type of problems is coordination problems that arise because sequential multi-provider frameworks will typically introduce interdependencies (externalities and strategic incentives) and these coordination problems are addressed by introducing integrated penalties.

The set of cost-based contracts, however, will not produce the welfare-optimal solution, unless all providers are perfect agents, since the use of cost-sharing with necessity produces inadequate cost containment incentives. Hence, the sponsor, when designing the second-best contracts, must trade-off internal inefficiency concerns with concerns for inadequate quality provision. If the welfare loss that follows from introducing a pure prospective contract, arising from preference misalignments and poor coordination, is higher than the welfare loss that follows from introducing cost-based contracts, appearing as internal inefficiencies, the cost-based contracts become the socially preferred contracts. Furthermore, explicitly using ex post treatment cost information when practicing retrospective contracts will, most likely, reduce the level of provider payments (lower social cost) relative to the case of practicing prospective contracts.

Our findings are of importance in view of aging populations and the increase in the number of clients that need services from multiple providers. Our study also adds to the emerging literature on integrated care and integrated funding. This literature, however, is vague about the fundamental sources to uncoordinated care. In most cases, authors tend to refer to symptoms, rather than the underlying mechanisms. In view of this, there is clearly a demand for more research on the (i) fundamental causes to poor coordination, (ii) the significance of such problems, and (iii) the effectiveness of the various initiatives meant to reduce such problems. Our model is at the conceptual level, containing a restricted number of variables and interdependencies, thus future research on integrated care and integrated funding should focus on more detailed models when discussing optimal contract design.

In our study, the causal factors to uncoordinated care are; (i) institutional and financial fragmentation that give rise to non-internalized externalities, and, (ii) sequential decision structures that produce strategic incentives. The solution to the above problems is in accordance with the following fundamental economic principle; decision makers, when making decisions, should consider all costs and benefits that follow from their decision-making. When having limited access to detailed information about provider efforts and provider quality levels, the set of cost-based contracts presented in this work, where the payments to the upstream and midstream providers depend negatively on the treatment cost of their followers, build upon this principle. We believe that institutional fragmentation, often pointed out as a problem within social and health care supply chains, might be weakened by applying such cost-based contracts.

## Figures and Tables

**Fig. 1:**
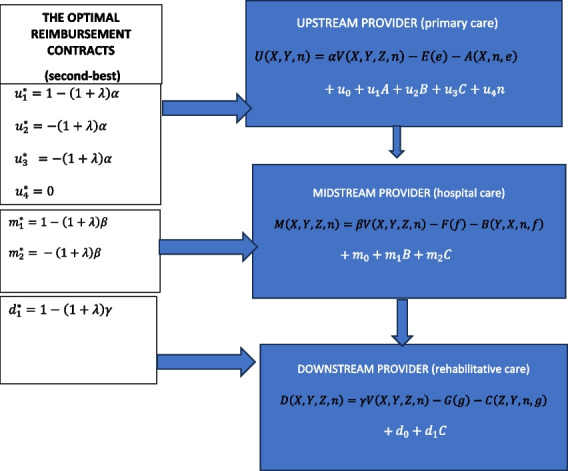
A flow diagram showing the relationships between three healthcare providers that organized sequentially and the corresponding cost-based reimbursement contracts

## Data Availability

No datasets were generated or analysed during the current study.

## Appendix 1

The derivatives of the response function can be easily described by assuming that the change in disutility that arises from an extra unit of cost containment, is independent of the other arguments of the cost function (${C}_{g}$ being independent of $(Y,X,n)$). If so, we arrive at the following derivatives for the downstream response function^37^:

$${z}_{Y}=-\frac{{D}_{YZ}}{{D}_{ZZ}}=-\frac{\gamma {V}_{ZY }-{C}_{ZY}}{\gamma {V}_{ZZ}-{C}_{ZZ}}$$

$${z}_{X}=-\frac{{D}_{XZ}}{{D}_{ZZ}}=-\frac{\gamma {V}_{ZX}}{\gamma {V}_{ZZ}{-C}_{ZZ}}$$

$${z}_{n}=-\frac{{D}_{nZ}}{{D}_{ZZ}}=-\frac{\gamma {V}_{Zn }-{C}_{Zn}}{\gamma {V}_{ZZ}-{C}_{ZZ}}$$

The denominator in the expressions above is strictly negative due to the second order condition, i.e., ${D}_{ZZ}=\gamma {V}_{ZZ}-{C}_{ZZ}<0$ and the signs of ${z}_{Y}$, ${z}_{X}$ and ${z}_{n}$ will depend on the signs of $\gamma {V}_{ZY}-{C}_{ZY}$, $\gamma {V}_{ZX}$, and $\gamma {V}_{Zn}-{C}_{Zn},$ respectively.

According to the terminology of Bulow et al., [12], the qualities *Z* and *Y* are strategical complements (strategic substitutes) when the downstream response function increases (decreases) with own quality, as midstream quality becomes higher, i.e., ${z}_{Y}\ge \left(<\right)0$. Hence, for both ${C}_{ZY}\le (>)0$ and ${V}_{ZY}\ge (<)0$, meaning a situation with decreasing (increasing) marginal downstream costs, as midstream quality becomes higher, in combination with the qualities being complements (substitutes) in the utility function, the qualities are strategical complements (substitutes). Generally, ${z}_{Y}$, could be both positive, zero and negative.^38^ Whether the downstream quality response function, from higher upstream quality and client group size, is positive or negative depends on the signs of ${z}_{X}$ and ${z}_{n}$. It is seen that if the qualities are complements (substitutes) in client utility, the downstream provider will increase (decrease) own quality when upstream quality is set higher, i.e. ${z}_{X}\ge \left(<\right)0$ as ${V}_{ZX}\ge \left(<\right)0$.^39^ Hence, in this case, since downstream costs are independent of upstream quality, the interaction between the two providers is solely dependent on the interdependencies between the qualities in the utility function. Moreover, it follows that when the utility of an extra client is increasing (decreasing) with higher downstream quality, ${V}_{Zn}\ge (<)0$, and the downstream marginal cost decreases (increases) with more clients, ${C}_{Zn}\le \left(>\right)0$*,* the downstream provider has an incentive to raise (reduce) quality in response to a higher number of clients, i.e. ${z}_{n}\ge \left(<\right)0$.

The midstream reaction function, $Y=y(X,n)$, is defined by (14a) and (14b). When ${B}_{f}$ is assumed independent of $(Y,X,n)$, the partial derivatives of this function simplifies to^40^:

$${y}_{X}=-\frac{{M}_{YX}}{{M}_{YY}}$$

$${y}_{n}=-\frac{{M}_{Yn}}{{M}_{YY}}$$

The denominator of the above expressions is negative from former assumptions, and is defined by ${M}_{YY}=\beta \left[{V}_{YY}+2{V}_{ZY}{z}_{Y}+{V}_{ZZ}{z}_{Y}^{2}+{V}_{Z}{z}_{YY}\right]-{B}_{YY}<0$. Hence, the signs of ${y}_{X}$ and ${y}_{n}$ are determined by the following two expressions:

$${y}_{X}\ge \left(<\right)0\ {M}_{YX}=\beta \left[{V}_{XY}+{V}_{YZ}{z}_{X}+{V}_{XZ}{z}_{Y}+{V}_{ZZ}{z}_{X}{z}_{Y}+{V}_{Z}{z}_{YX}\right]-{B}_{XY}\ge \left(<\right)0$$

$${{y}_{n}\ge \left(<\right)0\text{ as }M}_{Yn}=\beta \left[{V}_{Yn}+{V}_{YZ}{z}_{n}+{V}_{Zn}{z}_{Y}+{{V}_{ZZ}z}_{n}{z}_{Y}+{V}_{Z}{z}_{Yn}\right]-{B}_{Yn}\ge \left(<\right)0$$

From the above expressions it follows that whether *Y* and *X,* and *Y* and *n,* are strategic complements (substitutes), i.e., ${y}_{X}\ge \left(<\right)0$ and ${y}_{n}\ge \left(<\right)0$, will depend on the characteristics of three functions (clients’ benefits, the midstream costs, and the downstream reaction function). The first and the final terms in the expressions refer to the direct effect a change in the upstream provider’s choices have on the midstream provider’s quality, i.e. the ordinary effects $\beta {V}_{XY}-{B}_{XY}$ and $\beta {V}_{Yn}{-B}_{Yn}$. Additionally, when $X$ or $n$ changes, indirect effects occur since the downstream provider’s choice is affected, both from the upstream provider’s choice of $X$ or $n$, and from the midstream provider’s choice of $Y$, all influencing on the marginal gain or loss for the midstream provider to increase its choice of quality. These indirect effects are measured by the terms $\beta \left[{V}_{YZ}{z}_{X}+{V}_{XZ}{z}_{Y}+{V}_{ZZ}{z}_{X}{z}_{Y}+{V}_{Z}{z}_{YX}\right]$ and $\beta \left[{V}_{YZ}{z}_{n}+{V}_{Zn}{z}_{Y}+{{V}_{ZZ}z}_{n}{z}_{Y}+{V}_{Z}{z}_{Yn}\right]$.

## Appendix 2

The expressions for ${z}_{X}$, ${z}_{n}$, ${y}_{X}$ and ${y}_{n}$ in "Cost-based contracts" section are as follows:

$${z}_{X}=-\frac{{D}_{XZ}}{{D}_{ZZ}}=-\frac{\gamma {V}_{XZ}}{\gamma {V}_{ZZ}+{(d}_{1}-1){C}_{ZZ}}$$

$${z}_{n}=-\frac{{D}_{nZ}}{{D}_{ZZ}}=\frac{\gamma {V}_{Yn }-{{(d}_{1}-1)C}_{Yn}}{\gamma {V}_{ZZ}-{(d}_{1}-1){C}_{ZZ}}$$

$${y}_{X}=-\frac{{M}_{YX}}{{M}_{YY}}=\frac{\beta \left[{V}_{XY}+{V}_{XZ}{z}_{Y}+{V}_{Z}{z}_{YX}\right]+\left({m}_{1}-1\right){B}_{XY}}{\beta [{V}_{YY}+{2V}_{YZ}{z}_{Y}+{V}_{ZZ}\left({z}_{Y}^{2}+{V}_{Z}{z}_{YY}\right)]+\left({m}_{1}-1\right){B}_{YY}+{m}_{2}\left[{C}_{YY}+{C}_{YZ}{Z}_{Y}+{C}_{ZZ}{z}_{Y}^{2}+{C}_{YZ}{z}_{Y}+{C}_{Z}{z}_{YY}\right]}$$

$${y}_{n}=-\frac{{M}_{Yn}}{{M}_{YY}}=\frac{\beta \left[{V}_{Yn}+{V}_{Zn}{z}_{Y}+{V}_{Z}{z}_{Yn}\right]-\left({m}_{1}-1\right){B}_{Yn}}{\beta [{V}_{YY}+{2V}_{YZ}{z}_{Y}+{V}_{ZZ}\left({z}_{Y}^{2}+{V}_{Z}{z}_{YY}\right)]+\left({m}_{1}-1\right){B}_{YY}+{m}_{2}\left[{C}_{YY}+{C}_{YZ}{Z}_{Y}+{C}_{ZZ}{z}_{Y}^{2}+{C}_{YZ}{z}_{Y}+{C}_{Z}{z}_{YY}\right]}$$

The denominators, ${D}_{ZZ}$ and ${M}_{YY}$, are assumed to be negative. Generally, the signs of ${z}_{X}$, ${z}_{n}$, ${y}_{X}$ and ${y}_{n}$ are indetermined.

## Appendix 3

Figure 1 in Appendix 3 summarizes the main findings arrived at in “Cost-based contracts” section. The column at the left presents the optimal contract parameters while the right column presents the objective functions of the three providers that includes specifications of the cost-based contracts. It follows from the diagram that the optimal reimbursement scheme, besides the fixed budgets, consists of seven parameters of which six ($${u}_{1}^{*}, {u}_{2}^{*},{u}_{3}^{*} , {m}_{1}^{*}, {m}_{2}^{*} \text{and} {d}_{1}^{*})$$ are related to provider treatment costs while one is related to the number of treated patients ($${u}_{4}^{*})$$. It follows that structure of the contracts become less complicated as we move down the production chain.

The downstream provider is confronted with a contract that is contingent only upon downstream treatment costs, and this contract adjusts for imperfect downstream altruism and the cost of public funds (${d}_{1}^{*}$). This contract is a standard cost-sharing contract in the sense that it is only dependent on the treatment costs of the provider in question (own costs). ${d}_{1}^{*}$ can be both positive and negative depending on the degree of altruism held by the downstream provider.

The midstream provider is confronted with a contract which is dependent on both own costs ($${m}_{1}^{*}$$) and upon the treatment costs of the follower in the production chain ($${m}_{2}^{*})$$. As in the case of the downstream provider, the cost-sharing parameter related to own costs can be positive and negative, and this part of the contract adjusts for imperfect midstream altruism (preference alignment) and the cost of public funds. The second part of the contract of the midstream provider is contingent upon the treatment costs of the follower in the production chain, and this part is termed “an integrated penalty” since being strictly negative. This part of the contract is concerned with the coordination of midstream and downstream decision-making (the internalization of externalities and the neutralization of strategic incentives).

The upstream provider is confronted with an optimal contract that contains three elements. The fourth part of the contract, that relates to the number of patients, ${u}_{4}^{*}$, becomes equal to zero. The first of the three elements, ${u}_{1}^{*},$ is defining the (positive or negative) cost-sharing analogous to the similar elements commented on for the upstream and midstream provider. The second and third part of the contract, ${u}_{2}^{*}$ and ${u}_{3}^{*},$ are both strictly negative where ${u}_{2}^{*}$ is contingent upon the treatment costs of the midstream provider while ${u}_{3}^{*}$ is contingent upon the treatment costs of the downstream provider. Hence both these parts of the contract are, using our terminology, “integrated penalties” where ${u}_{2}^{*}$ is concerned with the coordination of upstream and midstream decision-making while ${u}_{3}^{*}$ is concerned with the coordination of upstream and downstream decision-making.

## Appendix 4

In the following, it is assumed that cost reducing effort and quality effort are part of the disutility function to each of the three providers, i.e.:

$$E=E\left(e,X\right), F=F(f,Y)\text{ and }G=G\left(g,Z\right), \text{where }{E}_{X}>0, {F}_{Y}>0\ \text{and } {G}_{Z}>0$$

This means that more quality increases both production costs and disutility costs. The welfare optimality conditions now become:

$${V}_{n}=\left(1+\lambda \right)\left[{A}_{n}{+B}_{n}{+C}_{n}\right], {V}_{X}=\left(1+\lambda \right)\left[{A}_{X}{+B}_{X}+{E}_{X}\right], -{A}_{e}={E}_{e}$$

$${V}_{Y}=\left(1+\lambda \right)\left[{B}_{Y}{+C}_{Y}+{F}_{Y}\right], -{B}_{f}={F}_{f}$$

$${V}_{Z}=\left(1+\lambda \right){[C}_{Z}+{G}_{Z}], -{C}_{g}={h}_{g}$$

Given the cost-based contracts, the behavior for the three providers become:

$${D}_{Z}=\gamma {V}_{Z}+{(d}_{1}-1){C}_{Z}-{G}_{Z}=0$$

$$-\left(1-{d}_{1}\right){C}_{g}={G}_{g}$$

$${M}_{Y}={\beta (V}_{Y}+{V}_{Z}{z}_{Y})+\left({m}_{1}-1\right){B}_{Y}+{m}_{2}\left({C}_{Z}{z}_{Y}+{C}_{Y}\right)-{F}_{Y}=0$$

$$-\left(1-{m}_{1}\right){B}_{f}={F}_{f}$$

$${U}_{X}=\alpha \left[{V}_{X}+{V}_{Y}{y}_{X}+{V}_{Z}\left({z}_{Y}{y}_{X}+{z}_{X}\right)\right]+\left({u}_{1}-1\right){A}_{X}+{u}_{2}{({B}_{Y}{y}_{X}+B}_{X})+{u}_{3}\left[{C}_{Z}{(z}_{Y}{y}_{X}+{z}_{X}\right)+{C}_{Y}{y}_{X}]-{E}_{X}=0$$

$${U}_{n}=\alpha \left[{V}_{n}+{V}_{Y}{y}_{n}+{V}_{Z}\left({z}_{Y}{y}_{n}+{z}_{n}\right)\right]+\left({u}_{1}-1\right){A}_{n}+{u}_{2}{({B}_{Y}{y}_{n}+B}_{n})+{u}_{3}\left[{C}_{Z}{(z}_{Y}{y}_{n}+{z}_{n}\right)+{C}_{Y}{y}_{n}+{C}_{n}]+{u}_{4}=0$$

$$-\left(1-{u}_{1}\right){A}_{e}={E}_{e}$$

It follows that the parameter values that eliminate preference misalignment and coordination problems (externalities and strategic effects) become as follows:

$${d}_{1}=\frac{[1-\gamma \left(1+\lambda \right)]({C}_{Z}+{G}_{Z})}{{C}_{Z}},{m}_{1}=1+\frac{1}{{B}_{Y}}\left\{{F}_{Y}+\beta \left(1+\lambda \right)\left[\frac{\left({C}_{Z}+{h}_{Z}\right){C}_{Y}}{{C}_{Z}}-\left({B}_{Y}{+C}_{Y}+{F}_{Y}\right)\right]\right\},$$

$${m}_{2}=-\frac{\beta \left(1+\lambda \right)\left({C}_{Z}+{G}_{Z}\right)}{{C}_{Z}}, {u}_{3}=-\frac{\alpha \left(1+\lambda \right)\left({C}_{Z}+{G}_{Z}\right)}{{C}_{Z}}, {u}_{2}=\frac{\alpha \left(1+\lambda \right)}{{B}_{Y}}\left(\frac{\left({C}_{Z}+{G}_{Z}\right){C}_{Y}}{{C}_{Z}}-\left({B}_{Y}{+C}_{Y}+{F}_{Y}\right)\right),$$

$${u}_{1}=1+\frac{1}{{A}_{X}}\{{E}_{X}-\alpha \left(1+\lambda \right)\left({A}_{X}{+B}_{X}+{E}_{X}\right)-\frac{\alpha \left(1+\lambda \right){B}_{X}}{{B}_{Y}}[\frac{\left({C}_{Z}+{G}_{Z}\right){C}_{Y}}{{C}_{Z}}-({B}_{Y}+{C}_{Y}+{F}_{Y})]\}$$

$${u}_{4}=\left(1+\lambda \right)\left[{A}_{n}{+B}_{n}+{C}_{n}\right]+\frac{\left(1+\lambda \right)}{{B}_{Y}}(\frac{\left({C}_{Z}+{G}_{Z}\right){C}_{Y}}{{C}_{Z}}-\left({B}_{Y}{+C}_{Y}+{F}_{Y}\right)){B}_{n}-\frac{\left(1+\lambda \right)\left({C}_{Z}+{G}_{Z}\right)}{{C}_{Z}}{C}_{n}+$$

$$\frac{1}{\alpha {A}_{X}}\{{E}_{X}-\alpha \left(1+\lambda \right)\left({A}_{X}{+B}_{X}+{E}_{X}\right)-\frac{\alpha \left(1+\lambda \right){B}_{X}}{{B}_{Y}}[\frac{\left({C}_{Z}+{G}_{Z}\right){C}_{Y}}{{C}_{Z}}-({B}_{Y}+{C}_{Y}+{F}_{Y})]\}{A}_{n}$$

As seen from above, the complexity, relative to the simplified model, now increases, implying that the optimal parameter values require a detailed knowledge of the functional relationships. It is also noted that the optimal value of ${\mu }_{4}$ now becomes different from zero.

## Appendix 5

Here we derive the optimal contract in the special case where the sponsor has verifiable information on the number of clients as well as the qualities supplied by the providers. Suppose now that the sponsor offers the following linear contracts to the providers:

$$u=u\left(X,Y,Z,n\right)={a}_{0}+{a}_{1}X+{a}_{2}Y+{a}_{3}Z+{a}_{4}n$$

$$m=m\left(Y,Z\right)={b}_{0}+{b}_{1}Y+{b}_{2}Z$$

$$d=d\left(Z\right)={c}_{0}+{c}_{1}Z$$

where ${a}_{j}, {b}_{i},{c}_{k},j=\text{0,1},\text{2,3},4, i=\text{0,1},2$ and $k=\text{0,1}$ are constants (parameters). By backward induction we arrive at the following optimality conditions (first the upstream provider chooses *X, n* and $e$, then the midstream provider chooses *Y* and $f,$ and finally the downstream provider chooses *Z* and $g$);

$$\gamma {V}_{Z}+{c}_{1}= {C}_{Z}$$

$$-{C}_{g}={G}_{g}$$

$$\beta \left({V}_{Y}+{V}_{Z}{z}_{Y}\right)+{b}_{1}+{b}_{2}{z}_{Y}={B}_{Y}$$

$$-{B}_{f}={F}_{f}$$

$$\alpha ({V}_{X}+{V}_{Y}{{y}_{X}+{V}_{Z}\left({z}_{X}+{z}_{Y}{y}_{X}\right))+a}_{1}+{a}_{2}{y}_{X}+{a}_{3}({z}_{X}+{z}_{Y}{y}_{X})={A}_{X}$$

$$\alpha ({V}_{n}+{V}_{Y}{{y}_{n}+{V}_{Z}\left({z}_{n}+{z}_{Y}{y}_{n}\right))+a}_{4}+{a}_{2}{y}_{n}+{a}_{3}({z}_{n}+{z}_{Y}{y}_{n})={A}_{n}$$

$$-{A}_{e}={E}_{e}$$

When comparing these conditions with the welfare optimal solution, we see that the parameters in the optimal contracts are set as follows:

$${a}_{1}={A}_{X}\left(1-\alpha \left(1+\lambda \right)\right)-\alpha \left(1+\lambda \right){B}_{X}, {a}_{2}=-\alpha {V}_{Y}, {a}_{3}=-\alpha {V}_{Z},$$

$${a}_{4}={A}_{n}\left(1-\alpha \left(1+\lambda \right)\right)-\alpha \left(1+\lambda \right){(B}_{n}+{C}_{n}),$$

$${b}_{1}={B}_{Y}\left(1-\beta \left(1+\lambda \right)\right)-\beta \left(1+\lambda \right){C}_{Y}, {b}_{2}=-\beta {V}_{Z},$$

$${c}_{1}={C}_{Z}\left(1-\gamma \left(1+\lambda \right)\right)$$

we arrive at the first best solution. Generally, the optimal contracts are then given by:

$$u={a}_{0}+\left[{A}_{X}\left(1-\alpha \left(1+\lambda \right)\right)-\alpha \left(1+\lambda \right){B}_{X}\right]X-\alpha {V}_{Y}Y-\alpha {V}_{Z}Z+{[A}_{n}\left(1-\alpha \left(1+\lambda \right)\right)-\alpha \left(1+\lambda \right){(B}_{n}+{C}_{n})]n$$

$$m={b}_{0}+\left[{B}_{Y}\left(1-\beta \left(1+\lambda \right)\right)-\beta \left(1+\lambda \right){C}_{Y}\right]Y-\beta {V}_{Z}Z$$

$$d={c}_{0}+{C}_{Z}\left(1-\gamma \left(1+\lambda \right)\right)Z$$

The optimal contracts are independent of realized costs, hence efficiency regarding cost reducing efforts is now secured. For the downstream provider, the contract is contingent upon own quality (see the last equation), thus being concerned with preference alignment. If $1>(\le )\gamma \left(1+\lambda \right)$, the payment to the downstream provider increases (decreases) with a higher quality level. For the midstream provider (see the second equation), the payment is dependent on own quality, *Y,* and the quality provided by the downstream provider, *Z*. The constant ${B}_{Y}\left(1-\beta \left(1+\lambda \right)\right)$ is concerned with preference alignment where $1>(\le )\beta \left(1+\lambda \right)$, contributes to higher (lower) payments as *Y* increases (decreases). The constant $-\beta \left(1+\lambda \right){C}_{Y}>0$ is concerned with the internalization of cost externalities and this effect is increasing with downstream quality. The last element of the contract i.e., $-\beta {V}_{Z}<0$, eliminates the strategic incentives faced by the midstream provider. For the upstream provider (see the first equation above), the structure of the optimal contract as concerning quality resembles that of the midstream provider. ${A}_{X}\left(1-\alpha \left(1+\lambda \right)\right)$ is concerned with preference alignment, $-\alpha \left(1+\lambda \right){B}_{X}$ ensures the internalization of externalities while $-\alpha \left({V}_{Y}+{V}_{Z}\right)<0$ eliminates the incentives to behave strategically. Finally, sponsor concerns with respect to client group size are addressed by ${A}_{n}\left(1-\alpha \left(1+\lambda \right)\right)-\alpha \left(1+\lambda \right){(B}_{n}+{C}_{n})$. In contrast to the case of cost-based contracts, the number of clients is now part of the optimal upstream contract.

## Footnotes

1 Social care services support people in dealing with the consequences of physical or cognitive impairment other than the (ongoing) health consequences [26].

2 The groups in question typically include vulnerable individuals such as drug abusers, individuals and families in acute distress psychiatric patients, elderly with high comorbidity, the terminally ill and patients with chronic diseases [87]. In the United States, 125 million people are living with chronic illnesses, disabilities, or functional limitations [7].

3 Retrospective contracts (cost plus contracts and cost-reimbursement contracts) typically refer to payment schemes where the providers are reimbursed after the services have been delivered. One example is when providers bill an insurance company for the actual costs incurred during the treatment of a patient. Cost-sharing refers to contracts where a share of the actual provider costs is shared with the sponsor. According to Mak [64], a defining characteristic of prospective payment, is that it pays providers irrespective of costs and quality. For a detailed discussion of retrospective and prospective contracts in health care see Jegers et al., [49].

4 The World Health Organization (WHO) defines care coordination as “a proactive approach to bringing together care professionals and providers to meet the needs of service users to ensure they receive integrated person-focused care across various settings ([88]; p. 8). Hughes et al., ([44], p.446) states that “integration is a broad concept, used to describe a connected set of clinical, organizational, and policy changes aimed at improving service efficiency, patient experience, and outcomes.

5 OECD [73] refers to the use of Best Practice Tariffs (BPT) and Integrated Care Systems in England and Accountable Care Organizations in the US as examples of more structurally integrated interventions. Other examples are The Better Care Fund and Care Trusts in England, Integrated Health and Social Service Boards in Northern Ireland and Social Health Maintenance Organizations in the US.

6 Implemented intersectoral interventions often include health and social care. According to Tebaldi and Stokes [82], an overarching element is the focus on preventive care and early intervention to reduce long-term health costs. However, sectors such as education, labor, agriculture, housing and environment may also be involved in intersectoral interventions (see [69] and [68]).

7 For the sake of simplicity, we ignore possible copayments that might affect utilization, and we do not discuss how income from such payments possibly reduces the need for public funding.

8 See for example Wolinsky [91], Matsumura and Matshushima [67], Lyon [61], Gravelle and Masiero [37], Nuscheler [72] and Karlson [52].

9 Our model also shares similarities with the literature on supply chains and value chains, as it involves multiple providers contributing to a final good. However, unlike supply chain literature, our model does not focus on the integrated processes between input and output producers, consumer demand in markets, or input-mix decisions within market equilibria. For example, such analyses are explored in recent studies by Li et al. [58] and Zhai et al. [93].

10 According to Arrow [2], the motives in health care differ from pure profit-maximization.

11 Wang et al. [86], specify the utility to be a Cobb-Douglas function with constant returns to scale of the following type, $U\left(B,\pi \right)={B}^{\alpha }{\pi }^{1-\alpha }$ where $\alpha$ is a measure of the relative weight of the patient benefit in the utility function. The estimates of the relative weight of the patient benefit are 0.51 (Chinese medical students), 0.42 (Chinese doctors) and 0.40 (German medical students.).

12 Godager and Wiesen [33] specify utility as $U\left(B,\pi \right)=\alpha B+\gamma \pi$ where $B$ is patients’ benefits and $\pi$ own profits where $\alpha$ and $\gamma$ are positive constants indicating the valuation of patient benefits and own profit, respectively. Their estimations show a median relative degree of altruism, $\alpha /\gamma$ , equal to 1,53 (mixed logit regressions) and 2,1 (multi-nominal logit regressions). The relative degree of altruism (the marginal rate of substitution) expresses how many units of profit the physician is willing to give up increasing the patients’ health benefits by one unit.

13 McGuire et al. [69] refers to studies from Kenya and Zambia where health care investments among school children were found to have positive effects on educational outcomes. Pakarinen et al. [74] finds that high-quality teacher-child interactions in kindergartens were positively associated with reading and math skills four years later.

14 Throughout the paper we have chosen to use the concept integrated penalty in such a case since a provider receives less the higher the cost of a subsequent provider becomes. An alternative would be to use the concept integrated reward, meaning that a provider is paid higher as the cost of a subsequent provider decreases.

15 More precisely, in our forthcoming analysis it is assumed that *V(X, Y, Z, n)* is strictly concavely increasing in *Y, X, Z* and *n*, i.e. that the Hessian of *V* is everywhere negative definite.

16 The provider cost structure matches an assumption of economics of scope, see Baumol et al. [4], Gravelle and Rees [36], Lipczynski et al., [59]. Here we do not discuss strategies of full integration.

17 One interpretation of the cost externalities is that subsequent providers change their optimal input mix in response to the quality provided by upstream providers.

18 To be precisely, we have in our analyses assumed that the *A*-, *B*- and *C*-functions are convex, i.e., that the Hessians of these functions are everywhere positive definite.

19 Given our assumptions concerning concavity and convexity of the basic functions above, the second order conditions will be satisfied.

20 The optimal transfers for the first-best solution are ${u}^{W}=A\left({X}^{W},{n}^{W},{e}^{W}\right)+E\left({e}^{W}\right)$, $m ^{W} = B \left({Y}^{W}, {X}^{W}, {n}^{W}, {f}^{W}\right) + F(f ^{W})$, ${d}^{W}=C\left({Z}^{W},{Y}^{W},{n}^{W},{g}^{W}\right)+G({g}^{W})$ and ${T}^{W}=A\left({X}^{W},{n}^{W},{e}^{W}\right)+E\left({e}^{W}\right)+B\left({Y}^{W},{X}^{W},{n}^{W},{f}^{W}\right)+F\left({f}^{W}\right)+C\left({Z}^{W},{Y}^{W},{n}^{W},{g}^{W}\right)+G({g}^{W})$.

21 It follows that the general prospective upstream contract is contingent upon client group size. Given this, a normative analysis could also be undertaken by optimizing with respect to ${\mu }_{1}$ since the size of this parameter, in addition to client group size, will have indirect effects on the provider quality variables and possibly cost-containment efforts.

22 We also observe, by comparing (12b), (13b) and (17b) with (9), (10) and (11), that prospective pay, also in the case of a sequential production chain, *ceteris paribus*, give the providers incentive to behave internally efficient.

23 Social inefficiency is here analyzed by comparing the optimality conditions with the welfare optimal conditions (the number and types of deviations). However, such an approach ignores that the degree of inefficiency (welfare sub-optimality) is the result of the distance between the various variables relative to the first best levels.

24 This observation points to the rationale for having a model with three providers rather than two.

25 In this case, we are left with the conditions described by (6–11) for a given client group size, $n=$ *n*.

26 Where $z_{\mathit y}=-\;D_{\mathit Y\mathit Z}/D_{\mathit Z\mathit Z}$ and $D_{ZZ}<0$ (to fulfill the second order condition).

27 This conclusion pre-supposes that the chain partners also provide the optimal quantities of quality and effort.

28 The expressions for ${z}_{X}$, ${z}_{n}$, ${y}_{X}$ and ${y}_{n}$ are available from Appendix 2. To determine whether the qualities are strategical substitutes or complements is more complex, relatively to the case with prospective contracts, since now depending, not only on the cross derivates of the cost functions and the benefit function, but also on the magnitude of effects when they work in opposite direction.

29 In Appendix 4, we extend our model to cover the case where providers, by increasing their quality, also are exposed to nonmonetary costs (disutility costs). It is shown that the main results remain, even though the determination of the parameter values that eliminate preference misalignment and coordination problems now becomes more complex. For instance, it is seen that now ${u}_{4}$ must be different from zero to internalize the production costs of the midstream, and the downstream provider experience if the upstream provider is going to choose the number of patients that is in accordance with the welfare optimal one.

30 Jack [48] employs standard techniques from the optimal regulation literature to examine the way a purchaser should optimally contract with providers with heterogenous and unknown degrees of altruism. He proposes a menu of non-linear contracts, relating payments to incurred costs, to induce physician to reveal their profile (ability and the degree of altruism).

31 This last finding has been attributed to extra administrative and management costs [46] and unmet needs [66].

32 Tebaldi and Stokes [82] differentiate between pooled approaches that target segments of the population and approaches that target broader geographically defined populations OECD [73] refers to the use of Best Practice Tariffs (BPT) as an example of bundling while Accountable Care Organizations (ACOs) in the US and Integrated Care Systems in England are mentioned as examples of population-based payments.

33 Examples are Care Trusts in England, Integrated Health and Social Service Boards in Northern Ireland and Social Health Maintenance Organizations in the US.

34 A more general set up would be to allow for the client group size to differ across providers. A possibility would be that the upstream provider supply services to a group of clients *n* where only some are accepted by the midstream provider, and finally, some of these again are accepted by the downstream provider. Adding waiting lists and queuing costs, causing externalities between the providers (and on the clients), add model complexity without necessarily providing additional insights.

35 Analyses concerned with accreditation incentives are available from Grepperud et al. [40] and Grepperud and Pedersen [39].

36 In Appendix 5, our model is analyzed for the case where the qualities are assumed to be contractible. The welfare-optimal contracts are defined by specific parameter values that depend on characteristics concerning the funding cost, the benefit function, the cost functions and the altruistic preferences of the providers. However, in practice, all dimensions of quality are difficult to observe and verify, leading to multitasking issues that are extensively discussed in the literature. Evidence related to pay-for-performance schemes confirms that the effects are rather modest, see for instance Eijkenaar [23] and Cashin et al., [14].
